# The impact of the COVID-19 epidemic on hospital admissions for alcohol-related liver disease and pancreatitis in Japan

**DOI:** 10.1038/s41598-021-92612-2

**Published:** 2021-07-12

**Authors:** Hisashi Itoshima, Jung-ho Shin, Daisuke Takada, Tetsuji Morishita, Susumu Kunisawa, Yuichi Imanaka

**Affiliations:** Department of Healthcare Economics and Quality Management, Graduate School of Medicine, Kyoto University, Kyoto, Yoshida Konoe‐cho, Kyoto 606‐8501 Japan

**Keywords:** Health policy, Public health

## Abstract

During the coronavirus disease 2019 (COVID-19) pandemic, there have been health concerns related to alcohol use and misuse. We aimed to examine the population-level change in cases of alcohol-related liver disease and pancreatitis that required admission during the COVID-19 epidemic by interrupted time series (ITS) analysis using claims data. We defined the period from April 2020, when the Japanese government declared a state of emergency, as the beginning of the COVID-19 epidemic. This ITS analysis included 3,026,389 overall admissions and 10,242 admissions for alcohol-related liver disease or pancreatitis from 257 hospitals between July 2018 and June 2020. The rate of admissions per 1000 admissions during the COVID-19 epidemic period (April 2020–June 2020) was 1.2 times (rate ratio: 1.22, 95% confidence interval: 1.12–1.33) compared to the pre-epidemic period. Analyses stratified by sex revealed that the increases in admission rates of alcohol-related liver disease or pancreatitis for females were higher than for males during the COVID-19 epidemic period. The COVID-19 epidemic in Japan might associates an increase in hospital admissions for alcohol-related liver disease and pancreatitis. Our study could support the concern of alcohol consumption and health problems during the COVID-19 pandemic.

## Introduction

Alcohol misuse is a major public health concern that causes about 3 million deaths worldwide each year^1^. During the coronavirus disease 2019 (COVID-19) pandemic, there have been health concerns related to alcohol use and misuse. Therefore, the World Health Organization cautioned that alcohol consumption during the pandemic might have a negative impact such as risk-taking behaviors, mental health problems and violence^2^. Stress is a risk factor for alcohol misuse. Policies such as keeping social distance and isolation could cause people stress. Therefore, it is recommended that governments give warnings about excessive alcohol consumption during isolation^3^.

Actually, since the stay-at-home policy began in some US states, one company has seen a 54% increase in national sales of alcohol per week, compared with 1 year before^4^. Some studies from the US and UK have reported an increased volume of alcohol consumption in households^5,6^. In Japan, a survey from the Japanese government reported that the expenditure in households for alcohol after April 2020 increased by 40–50% compared with 1 year before^7^.

Alcohol misuse also causes physical illness such as liver disorder and pancreatitis, alcohol-attributable fractions (AAAFs) for all global death, Disability-adjusted life years were accounted for 48%, 49% in liver cirrhosis and 26%, 28% in pancreatitis, respectively^1^. However, there have been few studies related to alcohol-related liver disease and pancreatitis during the COVID-19 pandemic.

This study examined the population-level change in cases of alcohol-related liver disease and pancreatitis that required admission during the COVID-19 epidemic.

## Results

### Main outcome

Overall hospital admissions were 3,026,389 cases, and 10,242 hospital admissions for alcohol-related liver disease or pancreatitis occurred in 257 hospitals, which had a total of 67,609 general beds during the study period. Of the 10,242 hospital admissions, 6371 cases were due to alcohol-related liver disease, and 3871 cases were for alcohol-related pancreatitis. Fourteen percent of alcohol-related liver disease cases had alcohol hepatitis, and 72% had alcoholic liver cirrhosis.

**Figure 1:**
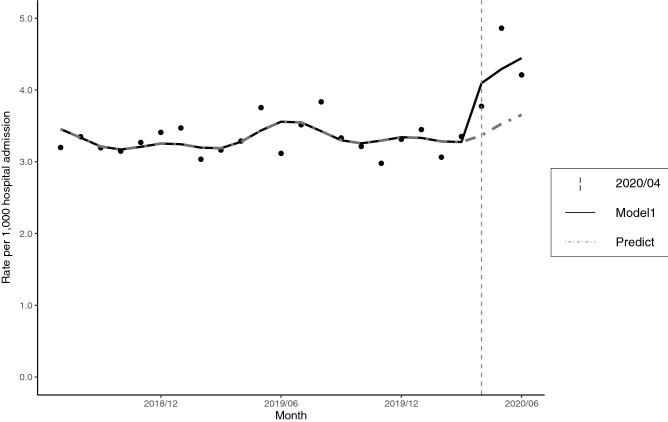
Hospital admissions for alcohol-related liver disease or pancreatitis during the study period. Model1: Poisson regression model, including trends and seasonality. Predict: Counterfactual scenario (if the epidemic did not occur).

The characteristics of the study population such as age, sex, length of hospital stay and in-hospital mortality did not change significantly between the pre-COVID-19 epidemic period (July 2018–March 2020) and the COVID-19 epidemic period (April 2020–June 2020) (Supplementary Table 1). The monthly number of hospital admissions with alcohol-related liver disease or pancreatitis, a year-on-year comparison and the monthly rates per 1000 hospital admissions were shown in Table 1. Figure 1 displayed these data together with the predicted regression curves. We observed an increase in the rate of hospital admissions with alcohol-related liver disease and pancreatitis immediately after the declaration of emergency for the COVID-19 epidemic by the Japanese government.

**Table 1:** Number of hospital admissions for alcohol-related liver disease or pancreatitis, and rates (cases/1000).

	Jul	Aug	Sep	Oct	Nov	Dec	Jan	Feb	Mar	Apr	May	Jun
**Total hospital admissions**												
July 2018–June 2019	140,400	137,665	123,972	139,791	134,314	128,476	138,884	125,211	129,625	130,147	123,317	88,234
July 2019–June 2020	142,223	135,317	129,970	136,624	132,632	134,289	137,757	125,029	129,761	109,718	94,209	78,852
Year-on-year (%)	101.30	98.29	104.84	97.73	98.75	104.52	99.19	99.85	100.10	84.30	76.40	89.37
**Alcohol-related liver disease or pancreatitis**												
July 2018–June 2019	448	457	395	440	438	434	479	377	409	427	459	275
July 2019–June 2020	497	513	430	439	393	444	474	381	433	414	456	330
Year-on-year (%)	110.94	112.25	108.86	99.77	89.73	102.30	98.96	101.06	105.87	96.96	99.35	120.00
**Rate per 1000 hospital admissions**												
July 2018–June 2019	3.19	3.32	3.19	3.15	3.26	3.38	3.45	3.01	3.16	3.28	3.72	3.12
July 2019–June 2020	3.49	3.79	3.31	3.21	2.96	3.31	3.44	3.05	3.34	3.77	4.84	4.19

The rate ratio (RR) for alcohol-related liver disease or pancreatitis during the COVID-19 epidemic period (April 2020–June 2020) compared with the pre-COVID-19 epidemic period (July 2018–March 2020) was 1.22 (95% confidence interval [95%CI] 1.12–1.33). Under the counterfactual scenario (if the COVID-19 epidemic did not occur), our model predicted 985.75 (95%CI: 968.38–1003.12) hospital admissions for alcohol-related liver disease or pancreatitis from April 2020 to June 2020, whereas 1,200 actual hospital admissions occurred during these three months. Therefore, this indicated that 214.25 (95%CI 196.88–231.62) excess hospital admissions took place during the COVID-19 epidemic period (Supplementary Table 2).

### Secondary outcomes and stratified analysis

**Figure 2:**
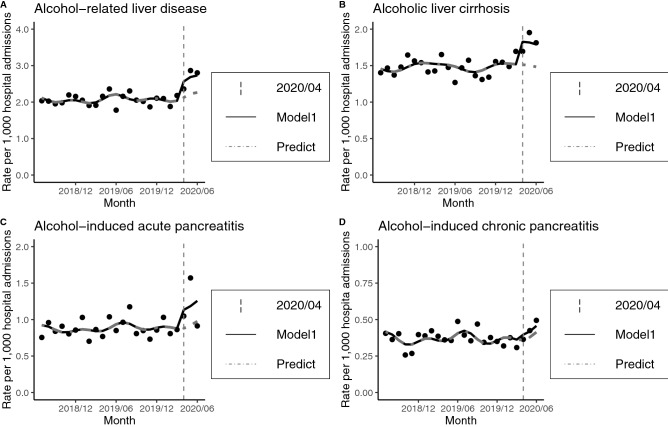
Hospital admissions for each disease during the study period. Model1: Poisson regression model, including trends and seasonality. Predict: Counterfactual scenario (if the epidemic did not occur).

Secondary analyses were performed for each diagnosis (alcohol-related liver disease, liver cirrhosis, acute pancreatitis and chronic pancreatitis). The monthly number of hospital admissions, year-on-year and the monthly rates per 1000 hospital admissions were displayed in Table 2. We displayed these data together with the predicted regression curves in Fig. 2. An increase in the rate of hospital admissions with each disease except for chronic pancreatitis was observed after the declaration of emergency for the COVID-19 pandemic by the Japanese government. Compared with acute pancreatitis cases, the monthly rates for cases of alcohol-related liver disease and liver cirrhosis gradually increased after the declaration of emergency. The RR for alcohol-related liver disease was 1.21 (95%CI 1.08–1.35). The RR for liver cirrhosis was 1.21 (95%CI 1.06–1.38). The RR for acute pancreatitis was 1.28 (95%CI 1.08–1.51). The RR for chronic pancreatitis was 1.10 (95%CI 0.84–1.44). The results for the impact of the COVID-19 epidemic were summarized in Table 3.

**Table 2:** Number of hospital admissions for each disease, and rates (cases/1000).

	Jul	Aug	Sep	Oct	Nov	Dec	Jan	Feb	Mar	Apr	May	Jun
**Alcohol-related liver disease**												
July 2018–June 2019	285	276	242	277	294	273	283	238	248	280	288	157
July 2019–June 2020	304	307	264	276	247	282	288	233	281	259	269	220
Year-on-year (%)	106.67	111.23	109.09	99.64	84.01	103.30	101.77	97.90	113.31	92.50	93.40	140.13
**Rate per 1000 hospital admissions**												
July 2018–June 2019	2.03	2.00	1.95	1.98	2.19	2.12	2.04	1.90	1.91	2.15	2.34	1.78
July 2019–June 2020	2.14	2.27	2.03	2.02	1.86	2.10	2.09	1.86	2.17	2.36	2.86	2.79
**Alcoholic liver cirrhosis**												
July 2018–June 2019	197	201	170	207	220	200	214	176	185	215	182	112
July 2019–June 2020	208	210	176	179	177	209	212	185	220	186	184	143
Year-on-year (%)	105.58	104.48	103.53	86.47	80.45	104.50	99.07	105.11	118.92	86.51	101.10	127.68
**Rate per 1000 hospital admissions**												
July 2018–June 2019	1.40	1.46	1.37	1.48	1.64	1.56	1.54	1.41	1.43	1.65	1.48	1.27
July 2019–June 2020	1.46	1.55	1.35	1.31	1.33	1.56	1.54	1.48	1.70	1.70	1.95	1.81
**Alcoholic acute pancreatitis**												
July 2018–June 2019	106	131	103	127	108	110	142	86	111	100	127	75
July 2019–June 2020	137	158	105	116	96	115	142	101	112	115	147	71
Year-on-year (%)	129.25	120.61	101.94	91.34	88.89	104.55	100.00	117.44	100.90	115.00	115.75	94.67
**Rate per 1000 hospital admissions**												
July 2018–June 2019	0.75	0.95	0.83	0.91	0.80	0.86	1.02	0.69	0.86	0.77	1.03	0.85
July 2019–June 2020	0.96	1.17	0.81	0.85	0.72	0.86	1.03	0.81	0.86	1.05	1.56	0.90
**Alcoholic chronic pancreatitis**												
July 2018–June 2019	57	50	50	36	36	51	54	53	50	47	44	43
July 2019–June 2020	56	48	61	47	50	47	44	47	40	40	40	39
Year-on-year (%)	98.25	96.00	122.00	130.56	138.89	92.16	81.48	88.68	80.00	85.11	90.91	90.70
**Rate per 1000 hospital admissions**												
July 2018–June 2019	0.41	0.36	0.40	0.26	0.27	0.40	0.39	0.42	0.39	0.36	0.36	0.49
July 2019–June 2020	0.39	0.35	0.47	0.34	0.38	0.35	0.32	0.38	0.31	0.36	0.42	0.49

**Table 3:** Rates of hospital admission for alcohol-related liver disease and pancreatitis during the Epidemic period compared with Pre-epidemic period.

Disease	RR*	Lower 95%CI	Upper 95%CI	*P* value
Alcohol-related liver disease or pancreatitis	1.22	1.12	1.33	< 0.001
Alcohol-related liver disease	1.21	1.08	1.35	0.001
Alcoholic liver cirrhosis	1.21	1.06	1.38	0.005
Alcoholic acute pancreatitis	1.28	1.08	1.51	0.004
Alcoholic chronic pancreatitis	1.10	0.84	1.44	0.467

Results adjusted for trend and seasonality.

The epidemic period: April 2020–June 2020.

The pre-epidemic period: July 2018–March 2020.

*CI* confidence interval.

*Rate ratio: rate of hospital admission for each disease per 1000 hospital admissions.

The results of the stratified analysis were shown in Supplementary Table 3 and 4. These showed the rates of admissions for alcohol-related liver disease or pancreatitis increased in females than in males during the COVID-19 epidemic period. Differences in rates of hospital admissions between younger ($<$ 65 years) and older adults ($\ge$ 65 years) were not observed.

## Discussion

In this research, we found that the COVID-19 epidemic was associated with an increase in hospital admissions for alcohol-related liver disease and pancreatitis except for chronic pancreatitis.

The rate of hospital admissions per 1000 hospital admissions during the COVID-19 epidemic increased by 1.2 times (RR 1.22, 95%CI 1.12–1.33) compared with the pre-epidemic period for cases of alcoholic liver disease or pancreatitis. The COVID-19 epidemic caused about 214.25 excess hospital admissions for alcoholic liver disease or pancreatitis based on predictions from our model.

Our data indicated that the ratio of alcohol-related liver disease and pancreatitis was around 4:3 (around 200 cases of liver disease and150 cases of acute and chronic pancreatitis). Harmful alcohol consumption generally causes more cases of liver disease than pancreatitis. We outlined our hypothesis on the proportion of alcohol-related liver disease and pancreatitis in the aim of our study, which was to assess hospital admissions for these diseases. It is generally known that pancreatitis patients (especially acute pancreatitis patients) almost always require hospitalization as opposed to liver disease patients for whom hospital admission is not necessary. We also evaluated outpatients with alcohol-related liver disease and pancreatitis, and there were 8906 cases of liver disease and 357 cases of pancreatitis that were not hospitalized during the study period. Therefore, these outcomes support the observed proportion between alcohol-related liver disease and pancreatitis in our study.

Several studies on alcohol consumption have been published since COVID-19 was first reported^5,8–10^. However, few studies have examined the association between the pandemic and alcohol-related physical illnesses, such as liver disease and pancreatitis, as of November 10, 2020, including during the 2003 Severe Acute Respiratory Syndrome (SARS) pandemic. Among more than 800 Hong Kong residents who were exposed to the SARS pandemic in 2003, 4.7% of males and 14.8% of females who were current drinkers reported an increase in drinking one year after the SARS pandemic^11^. In addition, the risk of presenting symptoms of alcohol use disorders three years after the SARS pandemic was about 1.5 times higher for affected individuals, such as health care workers in Beijing who worked in quarantine or high-risk wards, compared with unexposed hospital workers^12^.

Reports of alcohol consumption during the COVID-19 pandemic have varied. Several studies reported that alcohol consumption increased, but other studies reported that alcohol consumption decreased due to fewer opportunities for eating out or shutdown of retailers^5,8–10^. In Japan, a survey reported that household expenditure for alcohol after April 2020 increased, while beer companies reported a decrease in sales due to a decrease in sales to the restaurant industry^13,14^. Our results also indicated that alcoholic liver cirrhosis accounted for about half of the increase in hospital admissions, which was similar to the increase from alcohol-related liver disease, especially when expressed as a rate per 1000 hospital admissions. Previous studies have reported a relationship between alcoholism and not only liver cirrhosis but alcohol-related liver disease as well. Therefore, the increase in hospital admissions due to alcohol-related liver disease and liver cirrhosis might suggest that alcohol consumption increased in certain high-risk groups rather than in the whole population^15,16^. News that alcohol consumption among particular populations such as alcoholics actually increased might support this hypothesis^17–20^.

Our exploratory analysis implied that hospital admissions for alcohol-related liver disease or pancreatitis in females might have occurred more frequently compared with males. Pollard et al. reported a significant increase of 0.18 days for heavy drinking (95% CI 0.04–0.32 days) in women in the US, which represents an increase of 41% from a 2019 baseline of 0.44 days^5^.

These results suggest that the COVID-19 pandemic negatively affected more females than males. Previous research shows a greater adverse effect of alcohol consumption on female health due to their different biological characteristics^21^. In addition, alcohol use disorder has been related to the stress caused by the economic crisis, such as job loss and decreased income^22^ and social isolation during lockdown^3,9^. Thus, females could be received greater impacts than males due to their increased exposure to the economic crisis during the COVID-19 pandemic. Loss of employment was globally reported to be 5.0% for women and 3.9% for men in 2020^23^. In contrast, the Labour Force Survey in Japan showed the unemployment rate was higher in men than in women^24^. However, women are more likely to be employed in part-time work than men. Another survey revealed a 50% reduction in the available days for female part-time workers, and as such, these women were categorized as a “substantially unemployed person” in the survey^25^. Generally, these substantially unemployed people are not counted as unemployed people in public surveys. Concerningly, the survey suggests that substantial unemployment was more common among women, and the authors speculated there were nine hundred thousand substantially unemployed females in Japan^25^. Therefore, females in Japan were more negatively affected by the economic crisis during the COVID-19 epidemic, which may have led to increased alcohol consumption and hospitalization for alcohol-related liver disease and pancreatitis.

These results suggest that clinicians and policymakers may need to consider medical and policy measures for alcohol misuse in high-risk groups.

Our study has some limitations. First, our study could not reveal a relationship between individual alcohol consumption and hospital admissions for alcohol-related liver disease or pancreatitis. Therefore, further research is needed to confirm the association between alcohol consumption and hospital admissions for alcohol-related diseases. Second, our dataset did not include social or psychological factors. This point could be considered a limitation of our study; however, the impact of the COVID-19 pandemic is assumed to be an upstream factor contributing to the increased alcohol consumption resulting from psychological stress and the economic crisis^3,5,9,22,26^. Thus, we attest that this limitation was not crucial to our results, but further research is required to more directly assess the relationship between hospitalizations for alcohol-related liver disease or pancreatitis and socioeconomic status. Despite these limitations, the large sample size of our study is a key strength for assessing hospital admissions for alcohol-related liver disease and pancreatitis.

In conclusion, the COVID-19 epidemic might have increased the hospitalization rate for alcohol-related liver disease and pancreatitis due to an increase in alcohol consumption, particularly in females. Based on the results of this study, clinicians should be aware of the increase in alcohol-related admissions during the pandemic. Moreover, policymakers should keep in mind that measures such as lock-down for the pandemic might increase stress and result in hospitalization for alcohol-related liver disease and pancreatitis.

## Methods

### Study design

We conducted a quasi-experimental, interrupted time series analysis using Diagnosis Procedure Combination (DPC) data from the Quality Indicator/Improvement Project (QIP) database, which is administered by the Department of Healthcare Economics and Quality Management, Kyoto University. The QIP database consists of DPC data from acute care hospitals voluntarily participating in the project. The participating hospitals exceed 500, which are located throughout Japan and contain both public and private hospitals^27^.

In Japan, the DPC/pre-diem payment system (PDPS) is a prospective payment system that is accepted for acute care hospitals. The number of general beds adopted by the DPC/PDPS in 2018 accounted for 54% of total general beds in Japanese hospitals (482,618/891,872)^28,29^. The DPC data include insurance claims and clinical summary data, which contains facility identifiers, admission and discharge statuses (in-hospital death or alive discharge), cause of admission, primary diagnosis, the most and second -most medical resource-intensive diagnoses, up to 10 comorbidities and 10 complications. All diagnoses were classified according to the International Classification of Diseases, 10th Revision (ICD-10) codes. A published paper described further details^7^.

### Data collection

We included patients aged 18 years or older who were hospitalized between July 1, 2018 and June 30, 2020 from the database, and counted the admission cases whose both primary and most-medical resource-intensive diagnoses were alcohol-related liver disease or pancreatitis based on ICD-10 codes (K70·1: Alcoholic hepatitis, K70·2: Alcoholic fibrosis and sclerosis of the liver, K70·3: Alcoholic cirrhosis of liver, K70·4: Alcoholic hepatic failure, K70·9: Alcoholic liver disease, unspecified, K85·2: Alcohol-induced acute pancreatitis, K86·0: Alcohol-induced chronic pancreatitis). We defined K70·1, K70·2, K70·3, K70·4, and K70·7 as alcohol-related liver disease and K85·2, K86·0 as alcohol-related pancreatitis.

### Outcomes of interest

The main outcome was the rate ratio (RR) of hospital admissions with alcohol-related liver disease or pancreatitis per 1000 hospital admissions. Secondary outcomes were the RR of hospital admissions with alcohol-related liver disease, liver cirrhosis, acute pancreatitis and chronic pancreatitis per 1000 hospital admissions, respectively. In addition, excess hospital admissions for alcohol-related liver disease or pancreatitis were calculated.

### Statistical analyses

To compare year-on-year, we divided the study population into two: those who were admitted and discharged from July 2018 to June 2019 and from July 2019 to June 2020. Because the DPC data were generated on the day of discharge, no data were available for patients who had not been discharged by the end of June 2020, even if their admission date was before June 30, 2020.

We conducted an interrupted time series (ITS) analysis using segmented and Poisson regressions by considering seasonality, trends and overdispersion of data, to analyze the outcomes^30–32^. Seasonality was taken into consideration by adding harmonic terms (sines and cosines) with 12-month periods to our model^30^. The validity of the Poisson regression model was evaluated by using the correlograms (functions of autocorrelation and partial autocorrelation) and the residuals. Excess hospital admissions for alcohol-related liver disease or pancreatitis were defined as the difference in hospital admissions between the actual number of hospital admissions and the predicted number of admissions based on our model.

We defined the period from April 2020, when the Japanese government declared a state of emergency, as the beginning of the COVID-19 epidemic, and assumed that the COVID-19 pandemic rapidly affected the level of hospitalization after April 2020^33^. Several studies have found different alcohol consumptions by age (among younger and older adults) and sex^5,8^. Therefore, we conducted an exploratory stratified analysis for sex and cases aged below and above 65 years.

A two-sided *P* value < 0·05 was considered statistically significant, and all analyses were performed using R 3·6·3 (R Foundation for Statistical Computing, Vienna, Austria).

### Ethical considerations

We did not require informed consent because of the use of anonymized data, in accordance with the Ethical Guidelines for Medical and Health Research Involving Human Subjects (a provisional translation is available from: https://www.mhlw.go.jp/file/06-Seisakujouhou-10600000-Daijinkanboukouseikagakuka/0000080278.pdf), as stipulated by the Japanese Government. According to Part 12 section (1), (2) and (6) in this Guidelines, researchers may omit informed consent for a study that utilizes existing information. The present study was approved by The Ethics Committee, Graduate School of medicine, Kyoto University which waived the need of informed consent for the study (Approval Number: R0135).

All methods were performed in accordance with the relevant guidelines and regulations.

## Supplementary Information

Supplementary Tables.

## Data Availability

The datasets generated during and/or analyzed during the present study are available from the corresponding author on reasonable request.
